# Red Palm Oil Ameliorates Oxidative Challenge and Inflammatory Responses Associated with Lipopolysaccharide-Induced Hepatic Injury by Modulating NF-κβ and Nrf2/GCL/HO-1 Signaling Pathways in Rats

**DOI:** 10.3390/antiox11081629

**Published:** 2022-08-22

**Authors:** Olawale R. Ajuwon, Jeanine L. Marnewick, Oluwafemi O. Oguntibeju, Lester M. Davids

**Affiliations:** 1 Redox Biology Research Laboratory, Department of Biochemistry, Federal University Oye-Ekiti, Oye-Are Road, P.M.B. 373, Oye-Ekiti 371104, Nigeria; 2 Applied Microbial and Health Biotechnology Institute, Cape Peninsula University of Technology, Bellville 7535, South Africa; 3 Phytomedicine and Phytochemistry Group, Department of Biomedical Sciences, Faculty of Health and Wellness Sciences, Cape Peninsula University of Technology, Bellville 7535, South Africa; 4 Graduate School of Business, University of Cape Town, Cape Town 7700, South Africa

**Keywords:** antioxidant, lipopolysaccharide, red palm oil, oxidative stress, inflammation

## Abstract

Lipopolysaccharide (LPS), a well-conserved cell wall component of Gram positive bacteria, exerts its toxic effects via inducing oxidative and pro-inflammatory responses. Red palm oil (RPO) is a unique natural product with a balanced ratio of saturated and unsaturated fatty acids, with reported antioxidant and anti-inflammatory effects. In this study, we assess the protective effect and mechanistic action of RPO using a lipopolysaccharide (LPS)-induced hepatic injury model. Male Wistar rats were assigned into four groups (10 animals/group): normal control (NC), RPO, LPS and RPO + LPS. Animals in the RPO and RPO + LPS groups were administered RPO (200 μL/day) for 28 days. On the 27th day of experiment, animals in LPS and RPO + LPS groups were injected with LPS (0.5 mg/kg body weight). Animals were sacrificed 24 h later, and blood and liver tissues harvested for biochemical and molecular analysis. RPO resolved hepatic histological dysfunction induced by LPS, and lowered alanine aminotransferase, aspartate aminotransferase, alkaline phosphatase and γ-glutamyl transferase activities in the serum. Hepatic malondialdehyde and conjugated dienes, as well as pro-inflammatory cytokines, including interleukin (IL)-1β, IL-6 and TNFα were significantly diminished (*p* < 0.05) by RPO pre-treatment. Activity of hepatic antioxidant enzymes including superoxide dismutase, glutathione reductase, glutathione peroxidase, as well as glutathione redox status (GSH:GSSG), and markers of antioxidant capacity that decreased as a result of LPS injection were improved by RPO pre-treatment. Mechanistically, RPO up-regulated mRNA expression of redox sensitive transcription factor Nrf2 and its downstream targets GCL and HO-1, while also suppressing the expression of NFκβ and associated inflammatory protein, Iκβ kinase (IκKβ). In conclusion, this study highlights the ameliorating effects of RPO against LPS-induced hepatic injury and revealed the Nrf2/GCL/HO-1 and NFκβ signaling axis as potential contributing mechanisms.

## 1. Introduction

Lipopolysaccharide (LPS), a highly conserved cell wall component of Gram-negative bacteria triggers a systemic inflammatory response syndrome characterized by fever, hypotension, elevated heart rate, angioedema, intravascular coagulation, multiple organ failure and, in severe cases, septic shock, which is a major cause of mortality among patients in intensive care units all over the world [1]. The immune system in higher vertebrates identifies LPS as a pathogen-associated molecular pattern (PAMP), and its administration in vivo induces the classic inflammation model in multi-organs, including the liver, kidney and brain [2,3]. Due to its central role in the metabolism of xenobiotics, uniqueness in containing the largest population of tissue resident macrophages, anatomical localization and blood flow, the liver is particularly susceptible to injury by chemicals, including LPS. Exposure to LPS stimulate macrophages, especially Kupffer cells, and trigger the activation of a variety of transcription factors such as NF-κβ and activator protein-1 (AP-1). This ultimately results in the synthesis and release of inflammatory mediators including cytokines and chemokines (such as tumor necrosis factor-α (TNF-α), interleukin (IL)-1β, IL-6, IL-10 and vascular endothelial growth factor, VEGF), as well as increased expression of cyclooxygenase-2 (COX-2), inducible nitric oxide synthase (iNOS) and up-regulation of cell adhesion molecules (CAM) [4]. 

Beside inflammatory processes, oxidative stress has been suggested to be involved in the etiology of LPS-induced endotoxemia and organ damage. Over-production of COX-2 and iNOS generates reactive oxygen species (ROS), and reactive nitrogen species (RNS), such as superoxide anion radical (O_2_^•−^) and nitric oxide (NO), respectively [4]. The highly reactive O_2_^•−^ react easily with NO to produce peroxynitrite (ONOO^−^), another toxic mediator implicated in LPS-induced organ damage [5]. Besides their direct damage to the liver, free radicals may also induce the accumulation of leukocytes in the hepatocytes, causing further injury through activated neutrophils, which generate the enzyme, myeloperoxidase, and reactive oxygen species [6,7]. Excessive production of free radicals resulting from oxidative stress can lead to tissue injury via peroxidation of lipid membranes, protein oxidation and DNA damage. Lipid peroxidation induced by overproduction of ROS and RNS is believed to be an important cause of hepatic damage, with previous studies indicating that LPS alters cellular redox status by increasing malondialdehyde (MDA) and thiobarbituric acid reactive substances (TBARS) (both markers of lipid peroxidation), as well as depleting endogenous antioxidants such as the antioxidant enzymes and glutathione [8,9].

Regarded as a master regulator of redox homeostasis, Nrf2 (nuclear factor erythroid 2–related factor 2) is a transcription factor that coordinates the induction of varieties of cytoprotective proteins that play key roles in combating oxidative stress and chemical-induced toxic insults. Evidence has shown that Nrf2 responds to a series of cellular stress including redox alterations, proteotoxic stress, and nutrient deprivation to stimulate the expression of the wide range of genes involved in antioxidant and detoxifying mechanisms, such as heme oxygenase 1 (HO-1), glutamate cysteine ligase modifier subunit (GCLM), glutamate cysteine ligase catalytic subunit (GCLC), and NAD(P)H quinone oxidoreductase 1 (NQO1), among others [10,11]. An interplay between Nrf2 and NF-κβ has been suggested, with Nrf2 activation resulting in the production of downstream targets that have been reported to inactivate NF-κβ signaling, thus attenuating inflammatory responses [12,13]. Therefore, agents that can modulate the Nrf2-NF-κβ axis are now being explored as promising drug candidates in toxicant-induced organ damage where oxidative stress and inflammation are known to play significant role. 

Despite significant therapeutic and surgical advances, endotoxin-induced septic hepatic failure is still associated with significant deaths, making the development of a new therapeutic approach that could improve or complement existing treatment protocols an urgent need. In the last decade, there has been a tremendous increase in the use of whole plant extracts and/or isolated natural products [14]. The rise in popularity of these herbal medicinal products can be attributed to their being a cost effective alternative to orthodox treatments, as well as the assumption from anecdotal evidence that they are safe, with little or no toxicity, and that they have general public acceptance. Evidence has shown that whole plant extracts and isolated natural compounds possess potent biological effects, including cytoprotective, antioxidant, anti-inflammatory, anti-thrombotic, anti-mutagenic and tumor inhibitory effects. There are many reports highlighting the ability of several whole plant extracts, vegetables, teas and fruits, as well as isolated phytochemicals to activate Nrf2, modulates antioxidant response and attenuate the NF-κβ pathway [13,15,16,17,18].

Red palm oil (RPO) is a lipid product derived from the fleshy mesocarp of oil palm fruits. It is a unique oil, with an almost equal ratio of saturated and unsaturated fatty acids, and an excellent source of fat-soluble antioxidants such as the tocopherols, tocotrienols and carotenoids [19,20]. Animal model experiments indicated that RPO has many beneficial effects on health, including inhibiting oxidative stress [14,21], modulating lipid profile [22], and cardio-protection against ischemia-reperfusion injury [19]. In LPS-induced RAW264.7 macrophages, a tocotrienol-rich fraction of palm oil has been reported to demonstrate an anti-inflammatory effect by inhibiting the production of IL-6 and NO [23]. It is suggested that the cocktail of all antioxidant bioactive components found in RPO acts synergistically to bring about the observed health benefits. Based on these findings, we tested the hypothesis that dietary RPO supplementation for four weeks prior to LPS exposure will attenuate LPS-induced endotoxemia and hepatic damage in Wistar rats. We further aimed to elucidate the mechanism(s) involved. Results from our study suggest a scientific basis for the development of RPO as an adjuvant therapy in the treatment of LPS-induced hepatic damage.

## 2. Materials and Methods

### 2.1. Materials

The chemicals 5,5′-dithiobis-2-nitrobenzoic acid (DTNB), gallic acid, glutathione (reduced and oxidized), glutathione reductase (GR), 1-methyl-2-vinylpyridinium trifluoromethanesulfonate (M2VP) and β-nicotinamide adenine dinucleotide phosphate reduced tetrasodium salt (NADPH) were all products of Sigma–Aldrich (South Africa). Formaldehyde (histological grade), lipopolysaccharide (LPS; *Escherichia coli*, serotype 0111:B4), perchloric acid (PCA), 6-hydroxy-2,5,7,8-tetramethylchroman-2-carboxylic acid (trolox), 2-thiobarbituric acid (TBA) and trichloroacetic acid (TCA) were purchased from Sigma–Aldrich Chemicals, (St Louis, MO, USA). Ascorbic acid and malondialdehyde bis (diethyl acetal) (MDA) were products from Merck (Johannesburg, South Africa). Red palm oil (Carotino^TM^ baking fat) was a gift from Carotino SDN BHD (Johar-Bahru, Malaysia). All other reagents used were of analytical grade.

#### 2.1.1. Animals

Forty pathogen-free adult male Wistar rats weighing approximately 285 ± 13 g were used in the study. The rats were kept in the experimental animal holding facility at the Applied Microbial and Health Biotechnology Institute, Cape Peninsula University of Technology, South Africa and maintained under standard conditions of 21 ± 3 °C with 12 h light/12 h dark cycles, and humidity of 50 ± 5%. The rats were fed standard rat pellets (SRP) with access to clean tap water ad libitum.

#### 2.1.2. Ethical Approval

The animals were cared for humanely, and the study was conducted in accordance with criteria outlined in the National Institutes of Health Guide for the Care and Use of Laboratory Animals (NIH Pub. No. 85–23, Revised 1996). Ethical approval was granted by the Faculty of Health and Wellness Sciences Research Ethics Committee of the Cape Peninsula University of Technology, South Africa (Approval Number—CPUT/HW-REC 2011/A003).

### 2.2. Methods

#### 2.2.1. Induction of Hepatic Injury

To induce hepatic injury, overnight fasted rats were injected once via the intraperitoneal (i.p.) route with 0.5 mg/kg body weight of LPS (*E. coli* serotype 0111:B4) prepared in 10 mM PBS (pH, 7.2) [24]. 

#### 2.2.2. Study Design

Acclimatization in the animal holding facility lasted for 1 week, and rats were divided into four groups of 10 animals each, and treated for 4 weeks (28 days). The groups are normal control (NC group), RPO-treated control (RPO group), lipopolysaccharide-treated control (LPS group) and RPO-treated lipopolysaccharide group (RPO + LPS group). Animals in the NC and LPS group were fed only SRP, while those in the RPO and RPO + LPS group were fed RPO (200 µL, equivalent to 7 g/kg diet daily in the morning) in addition to the SRP. The selection of the RPO dose is based on previous work from our laboratory [14,25,26,27]. On the 27th day, animals in the LPS and RPO + LPS groups were injected intraperitoeally (i.p.) with 100 μL of lipopolysaccharide (equivalent to 0.5 mg/kg body weight) in PBS (10 mM, pH 7.2) to induce liver injury, while animals in the NC and RPO groups received 100 μL of the PBS vehicle. Throughout the study duration, feed intake, fluid intake, as well as general conditions of the animals were monitored. Body weights were recorded every week and on the day of sacrifice. At the end of the treatment period, overnight fasted animals were euthanized with sodium pentobarbital (100 mg/kg body weight, i.p.) and sacrificed. Blood samples were collected via the abdominal aorta and immediately centrifuged at 5000× *g* for 5 min at 4 °C to obtain serum. Separated serum was kept at −80 °C until used for various biochemical analyses. A portion of liver samples were removed and fixed in buffered formalin for histological analysis. The remaining liver samples were rinsed in ice-cold PBS (10 mM pH 7.2) to remove residual blood, blotted to dry, weighed and immediately frozen in liquid nitrogen, and stored at −80 °C for biochemical and molecular analysis.

#### 2.2.3. Histopathological Analysis

Formalin-fixed liver samples were rinsed in ascending ethanol concentrations, embedded in paraffin, sectioned (3–5 μm) with the aid of a sledge microtome and stained with hematoxylin and eosin (H and E). Stained tissue sections were examined by a pathologist who was unaware of the study protocols at the Department of Anatomy, Stellenbosch University, South Africa. 

#### 2.2.4. Preparation of Liver Homogenate

Liver tissues were homogenized (1:10, *w*/*v*) in a buffer system made up of 50 mM NaH_2_PO_4,_ 1 mM EDTA and 0.5% triton X (pH, 7.5), centrifuged at 10,000× *g* for 10 min at 4 °C and stored at −80 °C until used for further biochemical analysis. Protein content of homogenates were determined using the bicinchoninic acid (BCA) protein assay kits (Pierce, IL, USA), following manufacturer’s instructions. 

### 2.3. Biochemical Analysis

#### 2.3.1. Vitamin E and Carotenoids Content of RPO

Content of vitamin E and carotenoids in the RPO used in this study were determined by high performance liquid chromatography (HPLC) according to the method of Iqbal et al. [28] and Rautenbach et al. [29], respectively, as we have previously reported [14].

#### 2.3.2. Hepatic Antioxidant Capacity

To prevent protein interference, an equal volume of liver homogenate was precipitated in 0.5 M perchloric acid, and centrifuged (10,000× *g*) at 4 °C for 10 min [30]. Total antioxidant capacities of the protein-free extracts obtained from liver homogenate were determined as oxygen radical absorbance capacity (ORAC) [31], trolox equivalent antioxidant capacity (TEAC) [32], and ferric reducing antioxidant power (FRAP) [33]. 

#### 2.3.3. Markers of Hepatocyte Damage

The degree of hepatocyte damage was evaluated by measuring serum enzyme activities of alkaline phosphatase (ALP), alanine aminotransferase (ALT), aspartate aminotransferase (AST) and gamma-glutamyl transferase (γ-GT), and the level of albumin (ALB) and total bilirubin (TBIL), in an EasyRA automated clinical chemistry analyzer (Medica Corporation, Bedford, MA, USA). Standard diagnostic kits for all the analytes were also obtained from Medica Corporation, (Bedford, MA, USA).

#### 2.3.4. Estimation of Hepatic Lipid Peroxidation

The concentration of malondialdehyde (MDA) and conjugated dienes (CD) were determined in the liver as markers of lipid peroxidation. Malondialdehyde is the most abundant individual aldehyde product resulting from lipid peroxidation, and is usually used as an indirect index of oxidative stress. Hepatic MDA was determined using a high performance liquid chromatographic method described by Khoschsorur et al. [34], while hepatic CD was determined according to the method of Recknagel and Glende [35]. 

#### 2.3.5. Determination of Hepatic Glutathione

Hepatic glutathione status was determined using a commercial kit (Bioxytech GSH/GSSG-412^TM^; OxisResearch^TM^, USA), following the manufacturers protocol. The principle of the protocol is based on a method previously described by Tietze [36] in which the concentration of total glutathione (GSHt) and oxidized glutathione (GSSG) were determined separately. The concentration of reduced GSH is then obtained as a difference between total and oxidized GSH using the equation below.

$$\mathrm{Reduced}\;\mathrm{GSH}=\mathrm{GSHt}-2\left[\mathrm{GSSG}\right]$$

#### 2.3.6. Determination of Hepatic Antioxidant Enzymes Activity

The activities of catalase (CAT), superoxide dismutase (SOD), glutathione peroxidase (GPx) and glutathione reductase (GR) were determined in liver homogenates according to methods described by Aebi [37], Crosti et al. [38], Ellerby and Bredesen [39], and Staal et al. [40], respectively. 

#### 2.3.7. Analysis of Inflammatory Biomarkers

Homogenates for the determination of tumor necrosis factor–alpha (TNF-α), interleukin (IL)-1β, IL-6 and IL-10 were prepared (10%, *w*/*v*) in 10 mM phosphate buffered saline (pH 7.2), and centrifuged twice at 15,000× *g* for 10 min at 4 °C. Analyses were carried out blindly in multiscreen 96-well filter plates using customized rat cytokine kits (Merck Millipore, Burlington, MA, USA) on the Bio Plex bead array platform (Bio Plex Pro^TM^, Bio Rad Laboratories, Hercules, CA, USA) following the instruction of kit manufacturer. Bead acquisition and median fluorescence intensities analysis were carried out using the Bio-Plex Manager software version 4.1.1 (Bio Rad Laboratories, Hercules, CA, USA).

#### 2.3.8. Gene Expression Analysis by Real Time Quantitative Polymerase Chain Reaction (RT-qPCR)

Total RNA from rat liver was extracted using the RNeasy^®^ Mini Kit (Qiagen, Hilden, Germany) following manufacturer’s instructions. To remove DNA contamination, extracted total RNA was treated with DNase I before cDNA synthesis. Exactly 1 μg of RNA sample was used to synthesize cDNA by reverse transcriptase reaction using M-MLV reverse transcriptase kit (Promega, Madison, WI, USA). cDNA synthesis followed a two-step reaction condition of 70 °C for 5 min and 42 °C for 1 h. Expression of mRNA was quantified by real time-quantitative PCR (RT-qPCR) using a Faststart Universal SYBR Green Master Mix kit (Roche, Basel, Switzerland) on an Applied Biosystems PCR detection system (StepOnePlus^TM^, Thermo Fisher Scientific, Waltham, MA, USA), in accordance with the manufacturer’s protocols. The reaction conditions for PCR were as follows: 95 °C for 3 min, followed by 40 cycles of 15 s at 95 °C, 60 °C for 30 s and 72 °C for 30 s. The relative amount of cDNA was quantified using the comparative cycle threshold method (delta-delta (ΔΔ) CT method), and level of mRNA expression normalized to β-actin. Primer sequences (Integraed DNA Technologies) specific to rat cDNA used in the PCR experiment are listed in Table 1.

### 2.4. Statistical Analyses

Values were expressed as mean ± SD. Differences between groups mean were analyzed by one-way analysis of variance (ANOVA), followed by the Student–Newman–Keuls test for all pairwise comparisons. Where data were not normally distributed, the Kruskal–Wallis test was used to test for group differences. Results were considered statistically significant at *p* < 0.001, *p* < 0.01 and *p* < 0.05. All statistical analyses were carried out using GraphPad Prism version 5.0 (GraphPad Software, San Diego, CA, USA).

## 3. Results

### 3.1. Vitamin E and Cartenoid Content of Red Palm Oil

**Figure 1:**
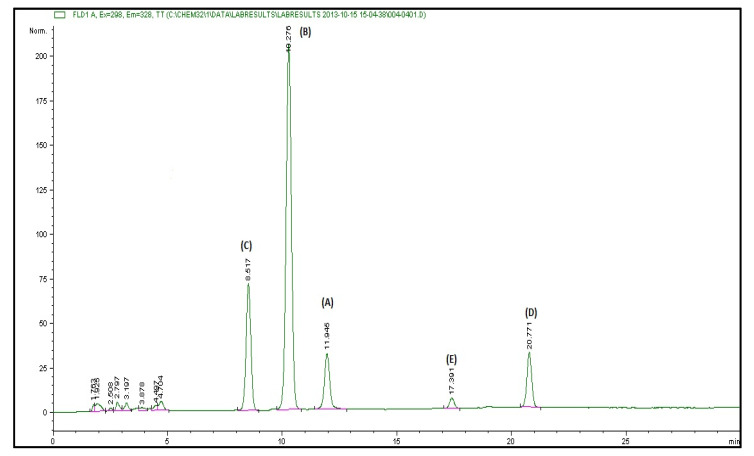
HPLC chromatogram showing different isoforms of vitamin E in the RPO used in the study (**A**) α-tocotriemol (**B**) β/γ-tocotrienol (**C**) δ-tocotrienol (**D**) α-tocopherol (**E**) δ-tocopherol.

The vitamin E and carotenoid content of the RPO used in this study is shown in Figure 1 and Table 2. The most abundant vitamin E isomers in the RPO are the tocotrienols accounting for about 80%. About 59% of carotenes in the RPO used in this study is β-carotene while the remaining 41% is made up of α-carotene. 

### 3.2. Weight Parameters, Vitamin E and Carotene Intake and Liver Antioxidant Capacity 

No death was recorded in the lipopolysaccharide-injected rat groups in the 24 h before sacrifice. Feed and water intakes were similar in all the rat groups (data not shown). The weight parameters as well as daily vitamin E and carotene intakes in the different experimental groups are reflected in Table 3. Body weight gain, liver weight and relative liver weight were not significantly different from each other in all the groups compared. Daily intakes of tocopherol (4.52 ± 0.15 vs. 4.63 ± 0.24), tocotrienol (18.02 ± 0.67 vs. 18.20 ± 0.94), α-carotene (1.09 ± 0.04 vs. 1.11 ± 0.06) and β-carotene (1.35 ± 0.04 vs. 1.38 ± 0.07) were similar between the two groups consuming RPO.

### 3.3. RPO Protects against LPS-Induced Hepatic Injury

**Figure 2:**
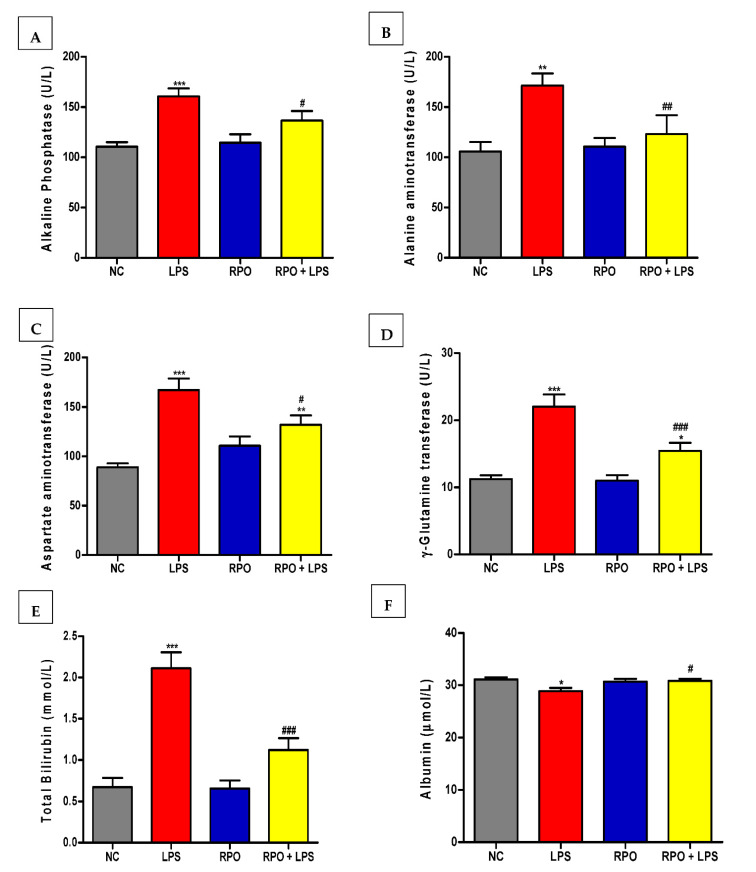
RPO prevents liver injury in LPS-induced rats. RPO ameliorated serum (**A**), alkaline phosphatase (**B**), alanine aminotransferase (**C**), aspartate aminotransferase (**D**), γ-glutamine transferase (**E**), total bilirubin and improved (**F**) albumin in rats injected with LPS. Data are mean ± SEM (*n* = 8–10). * *p* < 0.05, ** *p* < 0.01 and *** *p* < 0.001 as significant difference compared with NC. ^#^ *p* < 0.05, ^##^ *p* < 0.01 and ^###^ *p* < 0.001 as significant difference compared with LPS. NC (normal control group), LPS (lipopolysaccharide), RPO (red palm oil).

**Figure 3:**
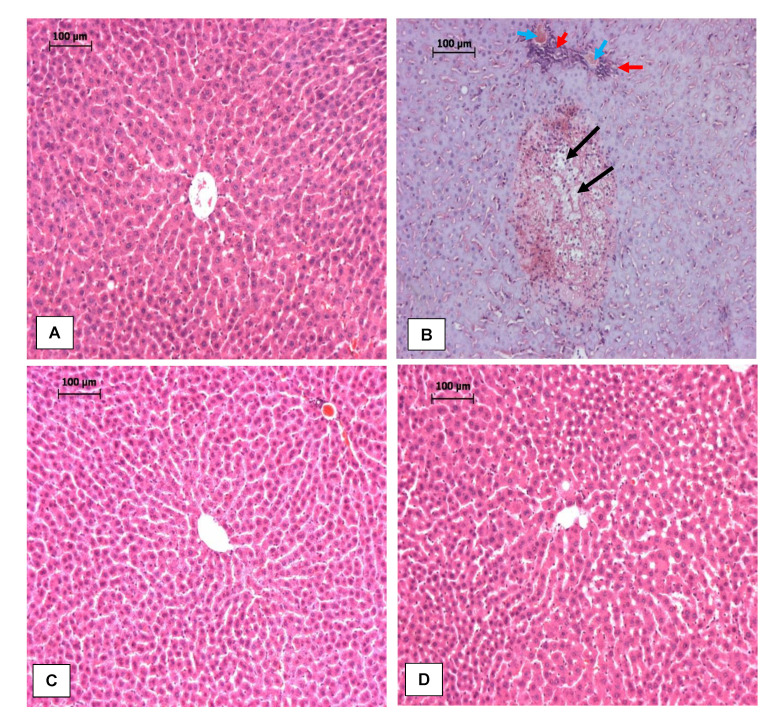
Photomicrograph of liver sections of (**A**) normal control rats showing normal liver architecture, (**B**) LPS-induced rats showing a large section of degenerative and necrotic changes (black arrows), leukocytes infiltration (red arrows) and hemorrhage (blue arrows), (**C**) RPO-treated rats with normal liver architecture and (**D**) LPS-induced rats pre-treated with RPO showing almost normal liver architecture. (Hematoxylin and Eosin (H&E); 100X).

As shown in Figure 2A–F, serum albumin (ALB), alkaline phosphatase (ALP), alanine aminotransaminases (ALT), aspartate aminotransferase (AST), γ-glutamine transferase (γ-GT) and total bilirubin (TBIL) were assayed to assess the protective effect of RPO on LPS-induced hepatotoxicity. LPS injection produced severe liver injury as shown by elevated level of the hepatocyte damage marker enzymes. Injection of LPS alone increased the level of ALP, ALT, AST, γ-GT and TBIL by 45%, 62%, 88%, 96% and 214%, respectively, when compared with negative control rats. LPS injection also induced a small but significant (*p* < 0.05) 7% reduction in serum albumin. Prior feeding of RPO for 4 weeks significantly lowers the elevation of the marker enzymes induced by LPS. The reduction amounted to 13%, 28%, 21%, 30% and 47% in ALP, ALT, AST, γ-GT and TBIL, respectively. RPO pre-treatment reversed the reduction in bilirubin significantly by about 7% (*p* < 0.05). Feeding RPO alone for 4 weeks did not induce any negative effect in the level of the hepatocyte damage marker enzymes. Examination of hematoxylin and eosin-stained sections revealed normal liver architecture in control (Figure 3A) and RPO-treated (Figure 3C) rats. LPS-induced rats exhibited hepatocyte degeneration, leukocyte infiltatration, necrosis and haemorrhage (Figure 3B). Rats that were pre-treated with RPO before LPS injection showed a marked improvement in morphological disruption with an almost normal liver architecture (Figure 3D).

### 3.4. RPO Attenuates Oxidative Stress, Enhances Antioxidant Enzymes and Restores GSH Redox Status in the Liver of LPS-Induced Rats

Since LPS-induced toxicity is associated with oxidative stress, we assessed the effect of RPO supplementation on hepatic antioxidant capacity measured as FRAP, ORAC and TEAC. As shown in Table 4, ORAC and TEAC values were similar across all experimental groups. However, LPS induced a significant (*p* < 0.05) reduction in antioxidant capacity measured as FRAP in the liver. Pre-treatment with RPO for 4 weeks ameliorated this reduction by returning the FRAP value to the level found in the normal control rats. Lipid peroxidation, activity of antioxidant enzymes and GSH redox status in the blood and hepatic tissue of LPS-induced endotoxemic rats were also measured. The extent of lipid peroxidation induced by LPS was evaluated by assessing the hepatic level of CD and MDA. LPS-induced oxidative stress resulted in a significantly (*p* < 0.05) higher level of CD and MDA in the liver, when compared to normal control rats. However, prior feeding of RPO for 4 weeks to LPS-injected rats significantly reduced the level of CD and MDA observed in the liver of the LPS-injected rats (Table 4). Regarding antioxidant enzymes, we observed that while the activities of SOD, GR and GPx were significantly reduced (*p* < 0.05) by LPS-injection, that of CAT was elevated in the endotoxemic rats. Pre-treatment with RPO reversed the observed effects by lowering the activity of CAT (22%) and increasing the activities of GR and GPx by 38% and 25%, respectively. Our study on the effect of RPO pre-treatment on glutathione redox status in endotoxemic rats showed that in the liver, GSH levels were similar in all the experimental groups, however, GSSG level was significantly elevated (*p* < 0.05) in the LPS-injected group compared to normal control rats. This subsequently resulted in a reduced GSH:GSSG ratio (13.64 ± 2.39 vs. 21.42 ± 6.32) in the LPS experimental group. RPO significantly (*p* < 0.05) increased the GSH:GSSG ratio by 37% in the LPS-injected rats, as shown in Table 4.

### 3.5. RPO Inhibits LPS-Induced NF-κβ Activation and Reduces Production of Inflammatory Cytokines 

**Figure 4:**
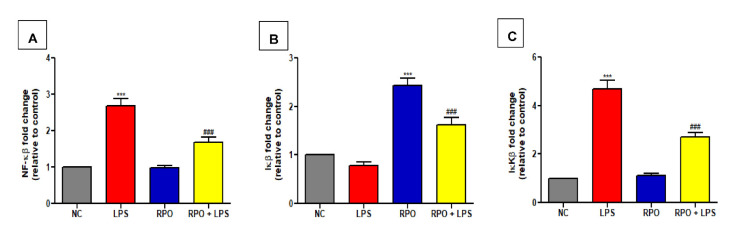
RPO inhibits NF-κβ activation in LPS-induced rats. RPO reduced hepatic (**A**) NF-κβ (**B**) Iκβ and (**C**) IκKβ gene expression. Data are mean ± SEM (*n* = 8–10). *** *p* < 0.001 as significant difference compared with NC. ^###^ *p* < 0.001 as significant difference compared with LPS. LPS (lipopolysaccharide), NC (normal control), RPO (red palm oil).

**Figure 5:**
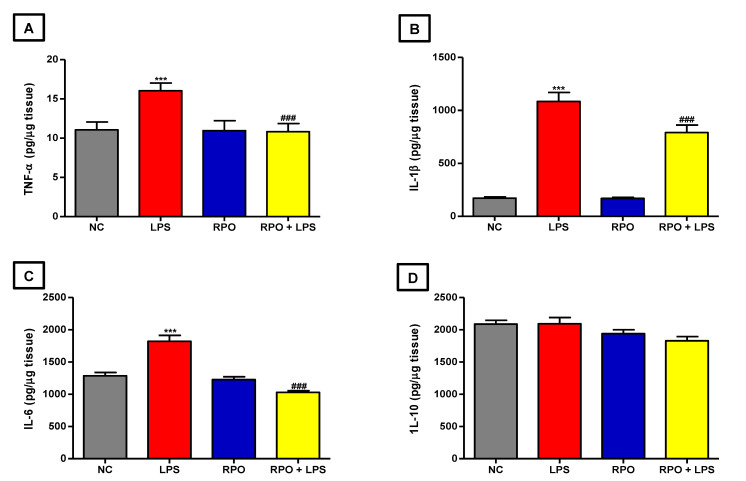
RPO suppresses release of pro-inflammatory cytokines in LPS-induced rats. RPO reduced (**A**) TNF-α (**B**) IL-1β (**C**) IL-6 and showed no effect on (**D**) IL-10 levels in the liver. Data are mean ± SEM (*n* = 8–10). *** *p* < 0.001 as significant difference compared with NC. ^###^ *p* < 0.001 as significant difference compared with LPS. LPS (lipopolysaccharide), NC (normal control), RPO (red palm oil).

NF-κβ as a redox-sensitive transcription factor regulates the formation of pro-inflammatory cytokines in response to increase oxidative stress. Therefore, we examined the effect of RPO pre-treatment on the mRNA expression of proteins in the NF-κβ signaling pathways in the liver of LPS-induced endotoxemic rats. Our results showed that LPS produced a 2.69-fold significant (*p* < 0.001) increase in NF-κβ mRNA expression in the liver when compared to control. RPO pre-treatment for 4 weeks caused a 1.60-fold significant (*p* < 0.001) decrease in LPS-induced NF-κβ mRNA expression in the liver (Figure 4A). We also observed that LPS injection caused a non-significant decrease in inhibitory kappa β (Iκβ) gene expression in the liver when compared to control. Rats consuming RPO alone showed a 2.43-fold significant (*p* < 0.001) increase in Iκβ mRNA expression when compared to negative control. Pre-treatment with RPO for 4 weeks was able to rescue the LPS-mediated reduction in Iκβ mRNA by significantly (*p* < 0.001) increasing its expression by 2.05-fold (Figure 4B). Furthermore, exposure to LPS significantly (*p* < 0.001) increased IκKβ (Iκβ kinase) mRNA expression in the liver by about 4.69-fold compared to negative control. RPO pre-treatment for 4 weeks significantly (*p* < 0.001) reduced LPS-induced expression of IκKβ by 1.73-fold (Figure 4C). The inflammatory response induced by LPS was further confirmed by a significant increase in the release of pro-inflammatory cytokines in the liver. LPS injection caused a 1.45- (*p* < 0.001), 6.32- (*p* < 0.001) and 1.42-fold significant increase in TNF-α, IL-1β and IL-6 levels, respectively, compared to control (Figure 5A–D), indicating the pro-inflammatory role of LPS in endotoxemic rats. However, pre-treatment with RPO significantly alleviated the pro-inflammatory effect of LPS. Specifically, RPO administration caused a significant 1.48 (*p* < 0.001), 1.37 (*p* < 0.001) and 1.77-fold (*p* < 0.001) decrease in TNF-α, IL-1β and IL-6 levels, respectively, when compared to non-pre-treated LPS-induced rats (Figure 5A–D). Our data indicate the possible involvement of the NF-κβ/Iκβ/IκKβ signaling pathway in LPS-induced inflammatory response, and that the anti-inflammatory effect of RPO may be exerted via this pathway. 

### 3.6. RPO Activates Nrf2 and Upregulates mRNA Expression of Downstream Targets HO-1 and GCL

**Figure 6:**
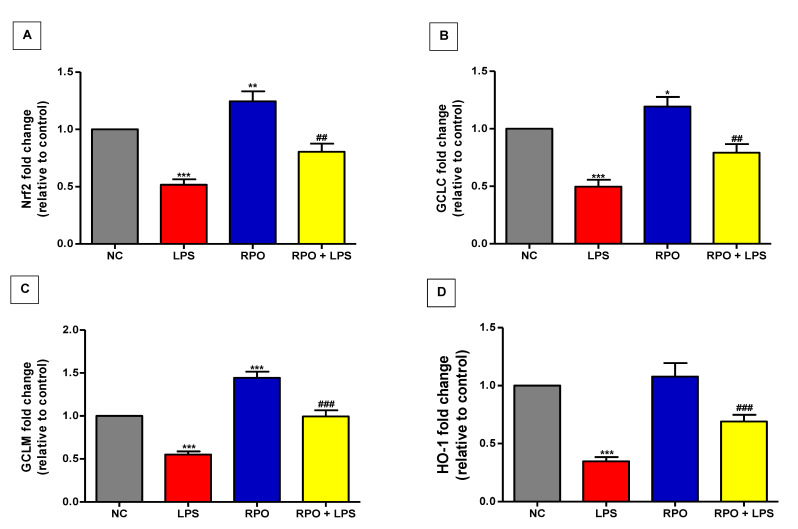
RPO activates Nrf2/HO-1/GCL signaling in LPS-induced rats. RPO increased (**A**) Nrf2 (**B**) GCLC (**C**) GCLM and (**D**) HO-1 gene expression in the liver of LPS-induced rats. Data are mean ± SEM (*n* = 8–10). * *p* < 0.05, ** *p* < 0.01 and *** *p* < 0.001 as significant difference compared with NC. ^##^ *p* < 0.01 and ^###^ *p* < 0.001 as significant difference compared with LPS. LPS (lipopolysaccharide), NC (normal control), RPO (red palm oil).

The activation of Nrf2 and induction of its downstream antioxidative and cytoprotective enzymes have been reported to play crucial role in the defense against LPS-induced hepatotoxicity. Consequently, we assessed the effect of LPS on gene expression of Nrf2 and downstream target enzymes GCLM, GCLC and HO-1 in this study. We also evaluated whether RPO pre-treatment for 4 weeks had any effect on the expression of these enzymes. Our results showed that LPS injection significantly (*p* < 0.001) diminished Nrf2 gene expression by about 48%. The suppressed Nrf2 signaling in LPS-intoxicated rats was confirmed by a significantly (*p* < 0.001) reduced GCLC, GCLM and HO-1 gene expression. Rats that were pre-treated with RPO for 4 weeks before LPS intoxication showed a remarkable 1.56- (*p* < 0.01), 1.59- (*p* < 0.01), 1.81- (*p* < 0.001) and 1.99-fold (*p* < 0.001) improvement in hepatic Nrf2, GCLC, GCLM and HO-1 gene expression, respectively, when compared with LPS-intoxicated rats without RPO pre-treatment. Of note, RPO pre-treatment alone showed a significant induction of the Nrf2 signaling with a concomitant increase in hepatic GCLC and GCLM gene expression (Figure 6A–D).

## 4. Discussion

Recently, there has been an increase in the number of studies suggesting the effectiveness of plant-derived bioactive compounds in ameliorating LPS-induced hepatic injury [7,8,13,41,42]. In this study, we investigated the effects of RPO pre-treatment on lipopolysaccharide-induced oxidative stress, inflammatory response, and their underlying mechanisms in rat liver. Our results demonstrated that the antioxidant and anti-inflammatory activity of RPO contributes to its protective effect against LPS-induced liver injury. RPO is an antioxidant-rich oil, containing many bioactive components, including tocopherols, tocotrienols, carotenes, squalene, lycopene and coenzyme Q10 [19]. We first carried out a high-performance liquid chromatographic (HPLC) quantification of the RPO used in this study, and our results showed the presence of isoforms of tocopherol and tocotrienol, α-carotene and β-carotene in a proportion similar to what has been previously reported [14,20]. The different bioactive components found in RPO are known to exhibit diverse health benefits and can serve antioxidant, anti-inflammatory, cardioprotective, as well as hepatoprotective functions under different pathological conditions.

LPS-induced endotoxemia is an established, valuable and reproducible model to induce acute liver injury in rodents. In the liver, LPS, released from Gram-negative bacteria cell wall, is known to induce an over-stimulation of the Toll-like receptor (TLR) signaling pathway, which promotes nuclear translocation of NF-κβ, switching on the transcription of pro-inflammatory cytokines such as IL-1β, IL-6 and TNFα, as well as reactive oxygen species (ROS). This oxidant/antioxidant imbalance and inflammatory response are the major cause of acute liver injury observed in rodent models of LPS-induced endotoxemia [43,44,45]. Consistent with previous studies that showed elevated levels of liver function marker enzymes in LPS-intoxicated rats [8,41,45,46], our results showed that lipopolysaccharide intoxication caused detrimental hepatic injury indicated by increased activities of ALT, AST, ALP, γ-GT and total bilirubin (TBIL) in the serum of the rats. These enzymes are cytosolic enzymes of the hepatocyte and the increase in their activities in the serum indicates a leakage in plasma membrane permeability, which in turn is associated with hepatocellular degeneration and necrotic changes in the liver [47]. Furthermore, we observed a significant decline in the serum albumin level in LPS-intoxicated rats; this, when taken together with the elevation in serum total bilirubin, are also commonly used markers of hepatocellular damage [48]. Histopathological analysis of the liver showed that LPS induced marked morphological disruptions including hepatocyte degeneration, leukocyte infiltration and necrosis. Pre-treatment with RPO reversed the LPS-induced effect on serum levels of ALT, AST, ALP, γ-GT, bilirubin, albumin and regressed the morphological changes observed in the liver of the rats, suggesting the membrane-stabilizing and potent hepatoprotective effect of RPO. We had previously reported a decrease in some hepatic injury markers including ALT, AST and LDH in the serum of tert-butyl hydroperoxide-induced rats pre-treated with RPO [19]. Other reports have demonstrated the ability of vitamin E, β-carotene and lycopene, all important constituents of RPO to ameliorate LPS- and other chemicals-induced hepatotoxicity in rodent models [46,49,50,51]. We can ascribe the ability of RPO to lower elevated liver function enzymes and resolve hepatic morphological disruptions observed in our study to the stabilizing effect of RPO and/or its bioactive components on hepatocytes plasma membrane. Another plausible reason may be the reversal of injury to hepatic tissues due to the stimulation of hepatocellular protein synthesis and the regeneration of hepatocytes [52]. 

On exposure to LPS, Kupffer cells are activated and are also able to recruit activated neutrophils and monocytes into the liver, thus promoting the formation of ROS by these phagocytes. These events eventually resulted in the formation of cytokines, including TNFα, IL-1β and IL-6, all early and important mediators of liver injury [41,53]. The ability of LPS to induce inflammation has been well documented, and the release of pro-inflammatory cytokines during LPS-induced endotoxemia is a well-known phenomenon [44,54]. The elevated level of hepatic TNFα, IL-1β and IL-6 in the LPS-treated rats observed in this study supports data from those previous studies. Interestingly, our results also indicated that pre-treatment with RPO for four weeks lowered the level of TNFα, IL-1β and IL-6 in the liver of the rats, demonstrating the potential anti-inflammatory effects of RPO. These results are similar to those from previous studies reporting on the anti-inflammatory activities of RPO and/or its bioactive components. Many in vitro studies have reported the ability of RPO or RPO-derived constituents such as different isoforms of tocotrienols, tocopherols, β-carotene and lycopene to reduce the formation of pro-inflammatory cytokines including TNFα, IL-1β and IL-6 [23,55,56,57,58]. Similarly, evidence has shown that the tocotrienol-rich fraction (TRF) of palm oil significantly hindered the formation of pro-inflammatory cytokines TNFα, IL-1β and IL-6 in ovalbumin-challenged asthmatic, and collagen-induced arthritis rats [59,60]. Furthermore, our lab and others have also previously demonstrated the anti-inflammatory potential of RPO in different animal models [25,61]. IL-10 is an anti-inflammatory cytokine that is responsible for the regulation of the innate immune response. Reported to be antagonistic to TNFα in its modulation of inflammatory response [62], IL-10 is known to suppress the production of TNFα and other pro-inflammatory cytokines by inhibiting their gene expression in monocytes, macrophages, neutrophils and natural killer (NK) cells [61,63]. Interestingly, our results showed a lack of substantial effect on hepatic IL-10 by either LPS or RPO pre-treatment, supporting a previous suggestion that the inhibition of pro-inflammatory cytokines observed with natural products such as RPO may not be connected with IL-10 activation [52]. 

Oxidative stress, a biochemical imbalance between oxidant formation and endogenous antioxidants, has been implicated as a major culprit in LPS-induced organ damage. LPS enhances the production of free radicals such as ROS and depletes cellular antioxidant levels, which ultimately leads to oxidative stress. During LPS-induced oxidative stress, ROS and other free radicals generated can attack nearby lipids, causing alterations in membrane structure and function and eventually leading to lipid peroxidation. Here, we observed an increase in lipid peroxidation, evidenced by the elevation in MDA and CD content in the liver of LPS-induced rats as previously reported by us and others [8,52,64]. SOD is the major enzyme involved in the spontaneous dismutation of superoxide anion (O_2_**^•−^**) to H_2_O_2_, which is subsequently degraded to water and oxygen by CAT. Both GPx and GR are key enzymes of the glutathione defense system. The decomposition of hydrogen peroxide and lipid hydroperoxides is catalyzed by GPx, with reduced glutathione (GSH) serving as a co-substrate. GSH is then regenerated from GSSG in another reaction catalyzed by GR at the expense of NADPH. The tripeptide GSH is the major intracellular thiol antioxidant and it is involved in the defense against oxidative stress by removing ROS and other free radicals. In agreement with previous studies that have demonstrated impairment in the hepatic antioxidant defense system [8,42,45,65], we observed a diminution of GSH/GSSG ratio, SOD, GPx, and GR activity, as well as an increase in CAT activity in the LPS-induced rats in this study. While the depletion in the activity of SOD, GPx and GR may be a result of their deactivation and the inability of the enzymes to neutralize production of ROS after LPS induction [66], the increase in activity of hepatic CAT observed in lipopolysaccharide-treated rats may be a compensatory response to the overproduction of H_2_O_2_ arising from SOD action [52]. More importantly, we found that pre-treatment with RPO reversed the LPS-induced impairment of hepatic antioxidant defense system, as MDA and CD elevation observed were reduced significantly, while the depletion of the GSH/GSSG ratio and inhibition of antioxidant enzyme activity was also reversed, indicating that RPO exhibits an antioxidative ability. Although, we did not measure the mRNA expression of SOD, CAT, GPx and GR in this study, these enzymes are encoded by genes that are all downstream targets of the transcription factor, Nrf2. Therefore, their modulation, consequent to RPO pre-treatment, may be due to the ability of RPO to activate the Nrf2 signaling pathway, and up-regulate the expression of genes encoding these antioxidant enzymes. There is abundance evidence of the antioxidant effects of RPO. Some reports have shown that RPO acts as a potent antioxidant as it is linked to an improved recovery and the protection of oxygen susceptible cells when exposed to oxidative stress [26,27,67]. We have also previously reported that RPO was able to inhibit lipid peroxidation and reverse dysfunction in enzymatic and non-enzymatic antioxidants in rats challenged with tert-butylhydroperoxide [14]. Furthermore, a recent randomized controlled study in humans has revealed that RPO supplementation for 8 weeks reduced erythrocyte MDA and urinary isoprostane levels in patients with HCV-related liver disease [67]. These results taken together demonstrated that RPO and/or its bioactive components is an effective free radical scavenger, and it ameliorated oxidative stress by preventing oxidative damage and elevating antioxidant enzymes activity.

To further clarify the molecular mechanism of the observed modulatory effect of RPO on the redox and inflammatory response in LPS-induced rats, we investigated the effect of RPO on the NF-κβ- and Nrf2-signaling pathways. Evidence exists to suggest a functional crosstalk between the Nrf2 and NF-κβ pathways, with both transcriptional factors reported to inactivate each other via protein–protein interactions or through secondary messenger effects at a transcription level [68,69]. The activation of NF-κβ, a master regulator of inflammatory response in the cell is triggered by increased oxidative stress. Evidence has shown that NF-κβ can be activated via two separate pathways: the canonical pathway with IL-1, TNFα and Toll-like receptor (TLR) cytokines acting as triggers, and the non-canonical pathway where other TNF-family cytokines, β-cell activating factor, and receptor activator of NF-κβ ligand served as triggers [18,70,71]. In the canonical pathway, oxidative stress induces the activation of IκKβ which causes phosphorylation of Iκβ and subsequent release and nuclear translocation of NF-κβ, leading to the transcription of pro-inflammatory mediators [72]. We observed in this study that LPS up-regulates the expression of IκKβ and NF-κβ genes, but down-regulates Iκβ gene expression, thereby triggering the activation and translocation of NF-κβ to the nucleus. This suggests that the pro-inflammatory effect of toxicants generally, and LPS in particular, is likely mediated via the canonical NF-κβ-signaling cascade, in accordance with reports from several earlier studies [18,73]. It is noteworthy in this study that RPO pre-feeding for four weeks inhibited the activation of NF-κβ signaling as indicated by a decrease in the gene expression of IκKβ and NF-κβ, parallel with an increase in Iκβ expression. The inactivation of NF-κβ as a result of RPO pre-treatment may thus be responsible for the reduced level of hepatic pro-inflammatory cytokines as discussed earlier in this study. 

Nrf2, a redox-sensitive transcription factor, coordinates the induction of detoxifying enzymes and antioxidant proteins that protect against excess ROS-induced oxidative damage. Under normal conditions, Nrf2 is usually sequestered in the cytoplasm with KEAP1 (Kelch-like ECH-associated protein 1). The activation of Nrf2 results in its translocation from the cytoplasm to the nucleus where it forms a heterodimer with the sMAF (small musculo-aponeurotic fibrosarcoma) protein. The Nrf2-sMAF complex subsequently interacts with the antioxidant response element (ARE) sequence to mediate transcription of several downstream targets, including heme oxygenase 1 (HO-1) and glutamylcysteinyl ligase (GCL), thereby protecting cells against oxidative injury induced by toxicants. As stated earlier, a functional crosstalk exists between Nrf2 and NF-κβ signaling, thus suggesting a link between antioxidant and anti-inflammatory effects of modulators of these two pathways. Evidence that Nrf2 activation reduces inflammation by inhibiting ROS generation and decreasing pro-inflammatory cytokines is abundant in the literature. Persistent inflammation has been reported in Nrf2-deficient mice [74,75]. Similarly, in LPS-induced macrophages, Nrf2 inhibits the mRNA expression of pro-inflammatory cytokines including TNFα, IL-1β and IL-6 by binding directly at the proximity of these genes, thereby preventing the recruitment of RNA polymerase II [76]. Furthermore, it has been documented that Nrf2 exerts its control over the NF-κβ pathway through its negative repressor KEAP1 [77]. KEAP1 has been reported to negatively regulate NF-κβ by stabilizing Iκβ (NF-κβ inhibitor) and preventing the degradation of IκKβ [77,78]. In this study, we tested the hypothesis that the inhibition of Nrf2 and its downstream targets is implicated in the observed LPS-induced hepatic injury, and that their up-regulation by RPO pre-treatment might play an important role in ameliorating oxidative stress and inflammation. Our results revealed that LPS administration down-regulates the induction of the Nrf2 gene and its regulated targets HO-1, GCLC (catalytic subunit) and GCLM (modulatory subunit) in the liver. Although this is contrary to role of ROS as an activator of the Nrf2 signaling pathway, the reduction in gene expression of Nrf2 and its downstream targets could be attributed to an excessive and sustained ROS level generated by LPS, as recently reported [45,79]. In support of this notion are studies showing that excessive and sustained ROS generated by different toxicants down-regulate Nrf2 and its downstream targets in the liver of rats [80,81,82]. Interestingly, our results showed that RPO pre-treatment leads to an increase in the expression of Nrf2, HO-1, GCLC and GCLM genes, indicating a reversal of LPS-induced oxidative injury in the liver of the rats. GCL is the enzyme that catalyzes the rate limiting step in the biosynthesis of glutathione. As a result of Nrf2 activation, gene expression of GCL is up-regulated, leading to an increased level of GSH and other GSH-dependent enzymes favoring a reducing environment that has been reported to inactivate NF-κβ, resulting in reduced inflammation [12,83]. HO-1 catalyzes the regulatory step during heme oxidative catabolism, leading to formation of free iron, carbon monoxide (CO) and biliverdin, which is immediately converted to bilirubin [84]. Both CO and bilirubin are potent bioactive molecules with reported antioxidant, anti-inflammatory and anti-apoptotic functions; thus HO-1 has been mentioned to play key roles in the pathophysiology and prevention of many diseases [85,86]. Further evidence has also shown that Nrf2-induced up-regulation of HO-1 can enhance the generation of anti-inflammatory cytokines, while suppressing formation of pro-inflammatory cytokines in different experimental models [13,87,88,89]. All these observations suggested that activation of Nrf2 and up-regulation of its downstream targets HO-1, GCLC and GCLM may be involved in the ability of RPO to protect against LPS-induced inflammatory response in rats. 

To the best of our knowledge, the current study is the first to report the ability of RPO to up-regulate the gene expression of Nrf2 and its downstream targets HO-1, GCLC and GCLM in a rat model of LPS-induced hepatic injury. However, some studies have reported up-regulated Nrf2 signaling following treatment with bioactive components found in RPO. A study by Kawata et al. [55] showed that β-carotene and lycopene, both bioactive compounds found in RPO, up-regulate the expression of HO-1 in RAW264.7 cells. Another study also reported that lycopene ameliorates LPS-induced liver injury in rats by promoting the expression of Nrf2 and HO-1, while inactivating NF-κβ/COX-2 signaling [46]. Using a house dust mite-induced asthma model in mice, Peh et al. [90] was able to similarly show that γ-tocotrienol, an isoform of vitamin E that is abundant in RPO, was effective in preventing human dust mite-induced airway inflammation and oxidative stress by inhibiting NF-κβ nuclear translocation and enhancing Nrf2 levels in lung lysates. Furthermore, a study by Mishra et al. [17], combining wet lab and in silico experiments, revealed a possible activation of Nrf2 by vitamin E, either alone or in combination with curcumin in the heart of hypo- and hyper-thyroidic rats. Thus, we could attribute the ability of RPO to up-regulate Nrf2 and its downstream genes, as well inhibit NF-kβ activation to those bioactive compounds found in RPO.

It should be noted that we did not determine the effect of RPO pre-treatment on the protein expression levels of key proteins in the Nrf2/NF-κβ signaling axis. Therefore, we could not ascertain if the observed effect on their mRNA expression will correlate with their protein expression. We acknowledge that this may be an important limitation of this study that must be considered in future studies. 

## 5. Conclusions

**Figure 7:**
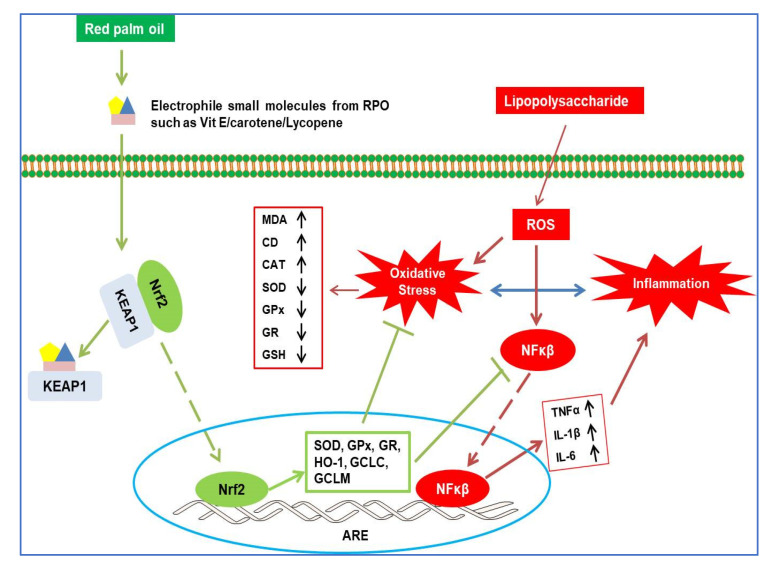
A proposed schematic representation showing the protective mechanism of red palm oil on LPS-induced hepatic injury. ARE, antioxidant response element; CAT, Catalase; CD, conjugated diene; GCLC, glutamate cysteine ligase catalytic subunit; GCLM, glutamate cysteine ligase modulatory subunit; GPx, glutathione peroxidase; GR, glutathione reductase; GSH, reduced glutathione; HO-1, heme oxygenase 1; IL-1β interleukin 1β; IL-6, interleukin 6; Keap1, Kelch-like ECH-associated protein 1; MDA, malondialdehyde; Nrf2, nuclear factor erythroid 2-related factor 2; NFκβ, nuclear factor kappa β; SOD, superoxide dismutase; TNFα; tumor necrosis factor α.

In conclusion, our results showed that in the liver of rats, RPO pre-treatment protects against LPS-induced oxidative stress and inflammatory responses. Administration of RPO attenuated liver damage, decreased MDA production and prevented the formation of pro-inflammatory cytokines (TNFα, IL-1β and IL-6). Similarly, depletion of antioxidant enzymes (SOD, GPx, and GR) and GSH imbalance was reversed by RPO pre-treatment. Mechanistically, our study provides new information that the antioxidant and anti-inflammatory effect of RPO is mediated, at least in part, via the inhibition of NF-κβ and the activation of Nrf2/GCL/HO-1 axis in LPS-induced rats (Summarized mechanistic pathways is presented in Figure 7). Despite containing an appreciable amount of saturated fatty acids, RPO is rich in natural fat-soluble carotenoids, tocopherols and tocotrienols (all reported to possess excellent antioxidant and anti-inflammatory properties), which may in part be responsible for the observed effects. Our findings support the use of a whole food-based approach for the prevention and treatment of pathological conditions in which oxidative stress and inflammation are known to play important role. However, future studies employing genetic and pharmacological approaches are recommended to further understand the role played by the Nrf2/GCL/HO-1 and NF-κβ signaling axis in the antioxidant and anti-inflammatory effect of RPO.

## Figures and Tables

**Table 1:** Primer sequence for real time quantitative PCR.

Gene	Sequence (5′->3′)
Nrf2 (NM_031789.3)	Forward: TGGATCTGTCAGCTACTCCCA Reverse: ATCCAGGGCAAGCGACTCAT
HO-1 (NM_012580.2)	Forward: CAGAAGGGTCAGGTGTCCAG Reverse: GAAGGCCATGTCCTGCTCTA
GCLC (NM_012815.2)	Forward: TCTGGATGATGCCAACGAGT Reverse: CCTCCATTGGTCGGAACTCT
GCLM (NM_017305.2)	Forward: CGCCTGCGGAAAAAGTGTC Reverse: TTCCACTGCATGGGACATGG
NFκβ (NM_199267.2)	Forward: CTGGCCATGGACGATCTGTT Reverse: GCACTTGTAACGGAAACGCA
Iκβ (NM_030867.2)	Forward: CCACTCCATGTAGCTGTCATC Reverse: ACACGTAGGCTCCGGTTTATT
IκKβ (NM_053355.3)	Forward: AGCTCTGGAACCTCCTGAAGA Reverse: CACTGGAAGGCTGGGACATT
β-actin (NM_031144.3)	Forward: CTCCCTGGAGAAGAGCTATGA Reverse: CAGGAAGGAAGGCTGGAAGA

Ascension number for each gene in parenthesis.

**Table 2:** Phytochemical and fatty acid constituent of RPO.

Constituents	Concentration
α-tocopherol (µg/g)	75.37 ± 10.50
β/γ-tocopherol(µg/g)	8.33 ± 1.05
δ-tocopherol (µg/g)	18.79 ± 4.80
α-tocotrienol (µg/g)	65.26 ± 6.43
β/γ-tocotrienol (µg/g)	242.21 ± 9.76
δ-tocotrienol (µg/g)	91.45 ± 5.94
α-carotene (µg/g)	20.44 ± 1.16
β-carotene (µg/g)	29.78 ± 2.92
SFA (%)	51
MUFA (%)	38
PUFA (%)	11

Values are mean ± SD of five determinations. SFA (saturated fatty acids), MUFA (mono-unsaturated fatty acids), PUFA (poly-unsaturated fatty acids).

**Table 3:** Effect of RPO supplementation on weight parameters, daily vitamin E and carotene intakes in LPS-induced rats.

Parameter	NC	LPS	RPO	RPO + LPS
Weight gain (g)	89.21 ± 17.31 ^a^	91.19 ± 11.57 ^a^	89.56 ± 5.09 ^a^	89.94 ± 7.93 ^a^
Liver weight (g)	11.83 ± 0.82 ^a^	12.36 ± 1.25 ^a^	11.83 ± 0.89 ^a^	12.06 ± 1.44 ^a^
Relative liver weight (%)	3.15 ± 0.22 ^a^	3.36 ± 0.43 ^a^	3.11 ± 0.28 ^a^	3.23 ± 0.27 ^a^
Intake of tocopherol/day (μg/100 g body weight)	Not determined	Not determined	4.52 ± 0.14 ^a^	4.63 ± 0.24 ^a^
Intake of tocotrienol/day (μg/100 g body weight)	Not determined	Not determined	18.02 ± 0.67 ^a^	18.20 ± 0.94 ^a^
Intake of α-carotene/day (μg/100 g body weight)	Not determined	Not determined	1.09 ± 0.04 ^a^	1.11 ± 0.06 ^a^
Intake of β-carotene/day (μg/100 g body weight)	Not determined	Not determined	1.35 ± 0.04 ^a^	1.38 ± 0.07 ^a^

Values are mean ± SD (*n* = 8–10). Similar alphabet in the same row showed no significant difference between groups (*p* > 0.05). LPS (lipopolysaccharide); NC (normal control); RPO (red palm oil).

**Table 4:** Effects of dietary RPO consumption on hepatic antioxidant capacity, lipid peroxidation, antioxidant enzymes activity and glutathione redox status in LPS-induced endotoxemic rats.

Parameters	NC	LPS	RPO	RPO + LPS
FRAP (μmol AAE/g tissue)	2.87 ± 0.25 ^a^	2.63 ± 0.13 ^b^	2.90 ± 0.17 ^a^	2.91 ± 0.18 ^a^
ORAC (μmol TE/g tissue)	22.14 ± 10.56 ^a^	17.40 ± 4.14 ^a^	19.61 ± 5.13 ^a^	17.72 ± 4.48 ^a^
TEAC (μmol TE/g tissue)	57.29 ± 3.48 ^a^	57.42 ± 4.37 ^a^	57.97 ± 9.02 ^a^	58.20 ± 4.45 ^a^
CD (nmol/g tissue)	10.38 ± 1.12 ^b^	12.78 ± 0.79 ^a^	11.15 ± 0.74 ^b^	11.12 ± 0.34 ^b^
MDA (µmol/g tissue)	64.83 ± 5.46 ^c^	92.65 ± 7.57 ^a^	70.62 ± 5.77 ^bc^	79.03 ± 13.91 ^b^
CAT (µmol H_2_O_2_ consumed/min/µg protein)	0.11 ± 0.01 ^c^	0.23 ± 0.03 ^a^	0.12 ± 0.03 ^c^	0.18 ± 0.02 ^b^
SOD (U/mg protein)	55.01 ± 5.27 ^a^	42.06 ± 6.26 ^b^	59.12 ± 13.82 ^a^	46.62 ± 7.09 ^b^
GR (µmol NADPH oxidized/min/µg protein)	3.99 ± 1.12 ^a^	2.69 ± 0.62 ^b^	4.10 ± 1.06 ^a^	3.71 ± 0.86 ^a^
GPx (nmol NADPH oxidized/min/µg protein)	0.16 ± 0.02 ^a^	0.12 ± 0.02 ^b^	0.17 ± 0.02 ^a^	0.15 ± 0.03 ^a^
GSH	6.38 ± 0.58 ^a^	6.37 ± 0.83 ^a^	6.50 ± 1.36 ^a^	6.20 ± 0.97 ^a^
GSSG	0.32 ± 0.10 ^b^	0.46 ± 0.09 ^a^	0.29 ± 0.04 ^b^	0.34 ± 0.05 ^b^
GSH:GSSG	21.42 ± 6.32 ^a^	13.64 ± 2.39 ^b^	23.09 ± 6.23 ^a^	18.72 ± 3.82 ^a^

Values are mean ± SD (*n* = 8–10). Different alphabet in the same row showed significant difference between groups at *p* < 0.05. AAE (ascorbic acid equivalent), CAT (catalase), FRAP (ferric reducing antioxidant power), GPx (glutathione peroxidase), GR (glutathione reductase), GSH (reduced glutathione), GSSG (oxidized glutathione), LPS (lipopolysaccharide), NC (normal control), ORAC (oxygen radical absorbance capacity), RPO (red palm oil), SOD (superoxide dismutase), TE (trolox equivalent), TEAC (trolox equivalent antioxidant capacity).

## Data Availability

Data are contained within the article.
